# Internal translation of Gja1 (Connexin43) to produce GJA1-20k: Implications for arrhythmia and ischemic-preconditioning

**DOI:** 10.3389/fphys.2022.1058954

**Published:** 2022-12-07

**Authors:** Claire C. Whisenant, Robin M. Shaw

**Affiliations:** Nora Eccles Harrison Cardiovascular Research and Training Institute, University of Utah, Salt Lake City, UT, United States

**Keywords:** GJA1-20k, Connexin43, mitochondria, ischemia, reperfusion, ischemic precondioning

## Abstract

Internal translation is a form of post-translation modification as it produces different proteins from one mRNA molecule by beginning translation at a methionine coding triplet downstream of the first methionine. Internal translation can eliminate domains of proteins that otherwise restrict movement or activity, thereby creating profound functional diversity. Connexin43 (Cx43), encoded by the gene *Gja1,* is the main gap junction protein necessary for propagating action potentials between adjacent cardiomyocytes. *Gja1* can be internally translated to produce a peptide 20 kD in length named GJA1-20k. This review focuses on the role of GJA1-20k in maintaining cardiac electrical rhythm as well as in ischemic preconditioning (IPC). Connexin43 is the only ion channel we are aware that has been reported to be subject to internal translation. We expect many other ion channels also undergo internal translation. The exploration of post-translational modification of ion channels, and in particular of internal translation, has the potential to greatly increase our understanding of both canonical and non-canonical ion channel biology.

## Introduction

Traditional eukaryotic ribosomal translation of an mRNA strand into a protein or peptide is cap-dependent, as it begins at the first AUG (methionine) start codon at the N-terminus (Basheer W. and Shaw R. 2016; Epifantseva and Shaw 2018). In addition to the start codon, most mRNA strands have AUG coding triplets downstream of the first methionine (Basheer W. A. and Shaw R. M. 2016). Internal translation by definition occurs when ribosomal translation of an mRNA strand begins not at the first start codon but at an internal ribosome entry site (IRES), which typically is at another (downstream) AUG triplet (Pestova et al., 1996; Epifantseva and Shaw 2018). Internal translation of an mRNA strand is therefore cap-independent and truncates the translated protein by omitting the N-terminal portion which is upstream of the internal methionine (Candeias et al., 2006; Basheer W. and Shaw R. 2016; Basheer W. A. and Shaw R. M. 2016). As a result of internal translation, a single strand of mRNA can potentially produce several different N-terminus truncated isoforms of the same protein, with the maximum number of isoforms produced equating to the number of downstream AUG triplets. Because internal translation is cap-independent, formation of the truncated isoforms is subject to different regulation than typical cap-dependent translation.

An mRNA strand is considered polycistronic when it can be translated into more than one peptide. Thus, like ubiquitination, phosphorylation, and methylation, internal translation is also a form of post-translational modification because it produces different peptides from one translated mRNA molecule. However, we urge the reader to consider the profound diversity introduced by internal translation. N-terminus truncations can eliminate membrane domains which otherwise restrict intracellular movement of peptides, as well as large conformational domains which otherwise inhibit biological activity of the remaining peptide (Candeias et al., 2006). It is not surprising that products of internal translation can have completely different biological functions than their full-length counterparts.

The potential of internal translation is especially applicable to ion channels. A truncated isoform of just the C-terminus of the channel without membrane domains will no longer need a lipid bilayer in which to exist or be transported. This type of truncated isoform will have greater mobility than its parent full-length channel. In addition, a truncated isoform of just the ion channel C-terminus will not be burdened by the physical presence of the larger transmembrane domains and structures, substantially increasing the likelihood of interaction with other proteins. This review considers what currently is known about a particular truncated isoform of the gap junction protein Connexin43, and how it both is a requisite auxiliary subunit of the larger channel necessary for gap junction trafficking and to prevent arrhythmia, and how it has multiple channel independent effects.

## Expression of GJA1-20k and role in full-length Cx43 trafficking

The gap junction alpha-1 (*Gja1*) gene encodes Connexin43 (Cx43), a gap junction protein necessary for propagating action potentials between adjacent cardiomyocytes to maintain cardiac rhythm as well as for cell cycle regulation, wound healing, and muscle differentiation (Shaw and Rudy 1997; Kardami et al., 2007; Vinken et al., 2012; Epifantseva and Shaw 2018). GJA1 mRNA is a polycistronic molecule (Smyth and Shaw 2013; Basheer W. and Shaw R. 2016). There are six downstream AUG (methionine) triplets after the initial methionine in the coding region of GJA1 mRNA (Smyth and Shaw 2013). Ribosomal translation can initiate at each of the methionines, producing the full-length 43 kDA protein (Connexin43), as well as truncated isoforms of approximately 32, 29, 26, 20, 11, and 7 kDA in size (Smyth and Shaw 2013). The 20 kDa isoform (GJA1-20k) is the predominant endogenous truncated isoform in human heart muscle (Smyth and Shaw 2013).

Internal translation is a phenomenon that is typically studied in the context of evolutionary changes in cells such as yeast (Ingolia et al., 2019). However, we are learning that internal translation is subject to metabolic pathway regulation, and thus is either turned off or on during different conditions of cellular stress (Basheer et al., 2018). For instance, the mammalian target of rapamycin (mTOR) pathway is involved with cardiovascular health and inhibition of this pathway is cardioprotective (Smyth and Shaw 2013). Cap-dependent translation is understood to be promoted by the mTOR pathway (Laplante and Sabatini 2009; Smyth and Shaw 2013). Inhibition of the mTOR pathway also increases GJA1-20k protein levels, supporting the mechanism of GJA1-20k translation as cap-independent and that increases in GJA1-20k may themselves be a mechanism of cardioprotection (Smyth and Shaw 2013; Basheer W. A. and Shaw R. M. 2016).

Full-length Cx43 must be efficiently delivered to the intercalated disc (ID) which is the communication foci between neighboring cardiomyocytes, to maintain intercellular electrical coupling and coordinated cardiac contraction. The half-life of Cx43 hemichannels is between two and five hours (Laird et al., 1991; Shaw et al., 2007). Due to this fast turnover rate, a targeted delivery mechanism is necessary by which full-length Cx43 is directly trafficked to the plasma membrane (Shaw et al., 2007; Smyth et al., 2010).

Targeted delivery is the paradigm by which specificity of ion channel delivery is due to a combination of a membrane anchor, the cytoskeleton delivery apparatus, and the particular channel being delivered (Shaw et al., 2007). GJA1-20k works with the cytoskeleton forward delivery apparatus to achieve targeted delivery (Basheer et al., 2017), explaining how the channel itself provides specificity which is *via* an auxiliary subunit which is a truncated isoform of the channel generated by the same mRNA. Evidence for the role of GJA1-20k in trafficking and targeted delivery is that when internal Cx43 isoforms of 32, 29, 26, and 20 kDa lengths are lost, full-length Cx43 is no longer delivered to IDs, resulting in poor cell-cell electrical coupling (Smyth and Shaw 2013). However, reintroduction of GJA1-20k without introducing other truncated isoforms restores full-length Cx43 to the ID (Smyth and Shaw 2013), indicating a role of GJA1-20k in trafficking Cx43 from the site of ribosomal translation at the endoplasmic reticulum/trans-Golgi network to the plasma membrane. Likewise, inhibiting the mTOR pathway, and thus increasing GJA1-20k expression by increasing cap-independent translation, causes an increase in Cx43 gap junction plaque size at the IDs of cardiomyocytes (Smyth and Shaw 2013), further supporting the role of GJA1-20k in trafficking Cx43 to the IDs. Site-directed mutagenesis of the internal AUG at codon 213 of GJA1 eliminates expression of GJA1-20k while retaining full-length Cx43 expression (Xiao et al., 2020).

A mouse model of GJA1-M213L results in mice with poor Cx43 expression at cell-cell borders of cardiomyocytes, electrograms classic for decreased cell-cell coupling, and mice that die suddenly between 2 and 4 weeks of age (Xiao et al., 2020). Without GJA1-20k, full-length Cx43 is not delivered to the intercalated disc and is more rapidly degraded, resulting in lower overall levels of Cx43 protein (Xiao et al., 2020). Expression of GJA1-20k is also decreased in hypertrophic cardiomyocytes, resulting in impaired trafficking of full-length Cx43 to the ID (Xiao et al., 2020; Fu et al., 2021). Overexpression of GJA1-20k by gene therapy limits ischemia related arrhythmias (Basheer et al., 2017).

## Ischemic preconditioning and I/R injury

Ischemic heart disease is the leading cause of death worldwide (Wong 2014), as occlusion of the coronary artery causes ischemic myocardium followed by necrosis (DeBoer et al., 1983). The duration of ischemia is directly correlated to the extent of necrotic myocardium (DeBoer et al., 1983). Rapid reperfusion of the occluded coronary artery is necessary to rescue oxygen-deprived cardiomyocytes before irreversible necrosis occurs (Reimer et al., 1977; DeBoer et al., 1983; Braunwald and Kloner 1985). However, reperfusion and rapid reestablishment of mitochondrial membrane potential causes a sudden increase in reactive oxygen species (ROS), leading to further oxidative stress and muscle damage (Griffiths et al., 1998; Halestrap et al., 2004; Basheer et al., 2018). ROS generation and damage post-reperfusion is known as ischemia/reperfusion (I/R) injury. As a result, there is a need to not only lessen the impact of original ischemia on cardiomyocytes, but also to avoid further I/R damage upon reperfusion.

Brief and repeated episodes of coronary occlusion prior to prolonged occlusion results in decreased infarct size (Murry et al., 1986). The phenomenon of lessening ischemic damage by preceding brief periods of ischemia is known as ischemic preconditioning (IPC). Despite 35 years of investigation, a definitive mediator of IPC protection has not been identified. There are multiple studies identifying mitochondria as central mediators of IPC. Mitochondrial protection can prevent metabolic failure and cardiomyocyte death (Gross and Auchampach 1992; Takashi et al., 1999; Opie and Sack 2002; Halestrap et al., 2004).

There exists strong associative evidence that Cx43 has a role in IPC protection (Garcia-Dorado et al., 1997). For instance, hearts and isolated cardiomyocytes from heterozygous Cx43-deficient mice cannot be preconditioned, whereas isolated cardiomyocytes from WT mice can be preconditioned (Li et al., 2002). As mitochondria have already been determined central to the mechanism of IPC, it is further hypothesized that this mechanism involves Cx43 at the mitochondria. This is supported by observed localization of Cx43 at the mitochondria using immunohistochemistry, and western blot evidence of Cx43 in mitochondria from isolated cardiomyocytes (Boengler et al., 2005). In addition, mitochondrial Cx43 is approximately doubled following IPC in a rat model of global ischemia compared to mitochondrial Cx43 in normoxic hearts (Boengler et al., 2005). Due to the short half-life of Cx43, it is hypothesized that this large increase of Cx43 in IPC is caused by new synthesis of Cx43, rather than inhibited degradation (Boengler et al., 2005).

It is not understood how Cx43, which is a hexameric ion channel each with four transmembrane domains and thus 24 transmembrane domains for each channel (Unwin and Zampighi 1980), either arrives at mitochondria or inserts in outer mitochondrial membrane. If Cx43 hemichannels translocate to the inner mitochondrial membrane *via* the TOM transporter to achieve IPC (Rodriguez-Sinovas et al., 2006), it is not clear how the hexameric 24 transmembrane domain channel is able to disassemble and unfold only to reconstitute as a functional ion channel in the inner membrane. It should be noted that antibody-based selection of Cx43 for the purposes of immunofluorescence and biochemistry involved Cx43 epitopes on the C-terminus which remains intact for many internally translated isoforms of Cx43 including GJA1-20k (Smyth and Shaw 2013).

## GJA1-20k in ischemic preconditioning and limiting I/R injury

A surprising finding is that when expressed in cells of different origin, GJA1-20k strongly colocalizes with mitochondria (Fu et al., 2017). Given the physical co-localization of GJA1-20k with mitochondria, the role of GJA1-20k was explored in the context of I/R injury and IPC. GJA1-20k increases 56% in *ex vivo* Langendorff-perfused mouse hearts subjected to I/R injury (Basheer et al., 2018). Likewise, GJA1-20k increases 5.3-fold in the heart after prolonged ischemic injury, modeled by 3-week occlusion of the left anterior descending (LAD) coronary artery in mouse hearts (Basheer et al., 2018). An increase in GJA1-20k is also seen in human heart samples from patients that suffered with chronic end-stage ischemic cardiomyopathy, as protein levels of GJA1-20k are increased 89% (Basheer et al., 2018). GJA1-20k is an apparently stress responsive protein increasing during acute and chronic ischemic stress.

**FIGURE 1:**
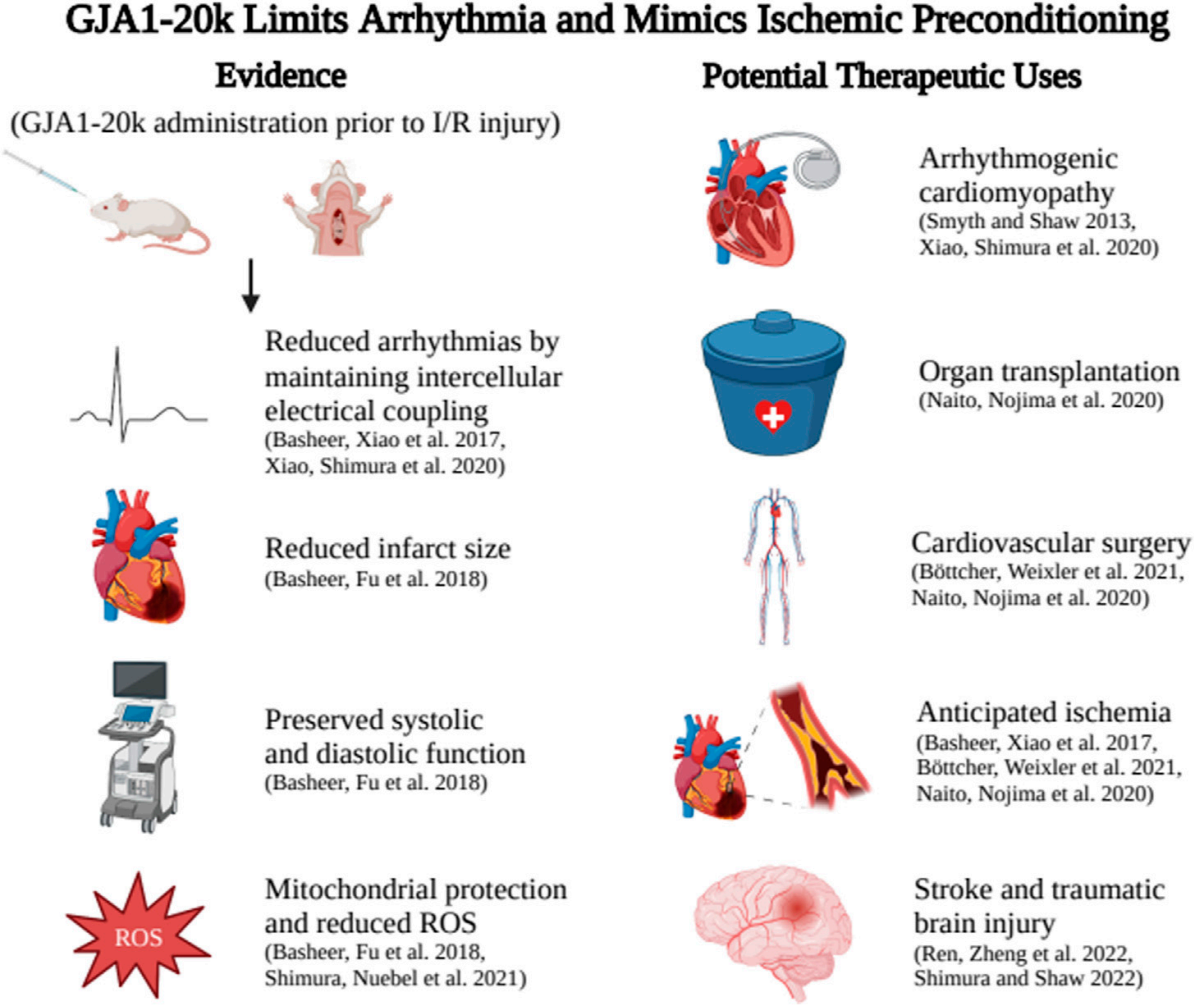
GJA1‐20k administration aids Connexin43 trafficking to reduce ventricular arrhythmia burden and also limits anticipated I/R injury by mimicking ischemic preconditioning. Potential therapeutic applications include reducing arrhythmia in genetic or acquired arrhythmia syndromes, preserving organs prior to transplant, pretreatment before angioplasty, cardiac surgery, or other situations of anticipated ischemia or use before anticipated brain injury to reduce the effects of stroke. Created with BIOrender.com.

As shown in Figure 1, introduction of exogenous GJA1-20k through an adeno associated viral vector (AAV9-GJA1-20k) before I/R injury in a mouse model significantly reduces infarct size, assessed 72 hours after LAD ligation, when compared to control littermates (Basheer et al., 2018). This indicates that treatment with GJA1-20k prior to I/R injury preserves cardiac muscle (Basheer et al., 2018). In addition, pretreatment with AAV9-GJA1-20k prevented the sharp elevation in left ventricular end diastolic pressure seen in control mice during the first 30 minutes of reperfusion after ischemic injury, indicating that treatment with GJA1-20k prior to I/R injury also is sufficient to preserve systolic and diastolic function (Basheer et al., 2018). In short, endogenous GJA1-20k increases with I/R injury and exogenous GJA1-20 k mimics IPC protection. These data highlight the potential of GJA1-20k as the much-sought mediator of IPC.

It should be noted that the increase in GJA1-20k after both prolonged ischemic injury and I/R injury is specifically localized to mitochondrial fractions, in or around the outer mitochondrial membrane (Basheer et al., 2018). The effect of GJA1-20k on mitochondrial membrane potential, respiration, and ROS production has also been investigated. Exogenous expression of GJA1-20k in mice hearts, achieved through an AAV9-GJA1-20k vector introduced to the venous system by retroorbital injection, induces metabolic quiescence, as indicated by a reduced mitochondrial maximal respiratory capacity, and reduced ROS production (Basheer et al., 2018). The metabolic quiescence is not unlike myocardial stunning which occurs after ischemic injury and itself is suggested to be protective (Ausma et al., 1998).

## GJA1-20k induces protective mitochondrial fission

As discussed above, GJA1-20k works with the actin cytoskeleton to facilitate full-length Cx43 delivery to the cell-cell border (Basheer et al., 2017). In cells and hearts, there is an inverse relationship between GJA1-20k and mitochondrial size, as an increase in GJA1-20k results in smaller mitochondria (Shimura et al., 2021). The cellular cytoskeleton is a candidate to mediate the interaction between GJA1-20k and mitochondria (Fu et al., 2017). Overexpression of GJA1-20k in HEK293 cells results in the formation of actin filament rings, recruited by GJA1-20k, around the outer membrane of mitochondria (Shimura et al., 2021). GJA1-20k recruited actin causes mitochondrial fission which is independent of the canonical mitochondrial fission mediator DRP1 and is also independent of MFN1 and MFN2 (Shimura et al., 2021).

It has been tested whether GJA1-20k mediated protective fission mechanism is central to IPC protection. A mouse model that lacks expression of GJA1-20k while maintaining expression for full-length Cx43 was originally generated to explore trafficking (Xiao et al., 2020) and used to explore the mechanism in IPC in I/R injury (Shimura et al., 2021). Heterozygous mice, which survive into adulthood with hearts that function similar to that of wild type mice, generate more ROS production following I/R injury and have massive infarctions relative to WT mice after I/R injury (Shimura et al., 2021). Endogenous GJA1-20k, upregulated during ischemia, appears to be highly protective against ischemic injury (Shimura et al., 2021) and exogenous GJA1-20k mimics IPC protection (Basheer et al., 2018). GJA1-20k is an attractive candidate to pharmaceutically mimic IPC protection.

### Potential roles for GJA1-20k therapy

Several studies highlight the potential of GJA1-20k as a therapeutic to both restore full-length Cx43 trafficking and protect against ischemic injury. Acute ischemia, modeled in mouse hearts subjected *ex vivo* to no-flow Langendorff-perfusion, results in reduced Cx43 localization at cell-cell borders, caused by interrupted trafficking of Cx43 due to actin disruption (Basheer et al., 2017). However, overexpression of GJA1-20k, introduced into mice through AAV9-GJA1-20k, restores Cx43 localization to cell-cell borders at a level similar to non-ischemic hearts (Basheer et al., 2017). Thus, pretreatment with GJA1-20k maintains Cx43 trafficking to cell-cell borders after ischemic injury despite actin disruption (Basheer et al., 2017).

Many ischemic events can be anticipated with opportunities to prevent ischemic injury. For example, major cardiovascular surgeries including repair of the ascending or abdominal aorta, coronary revascularization, and heart valve surgery may lead to ischemic injury of the heart, brain, and kidneys (Naito et al., 2020; Böttcher et al., 2021). In addition, organs harvested for transplantation including hearts, kidneys, livers, and lungs are all subject to ischemic injury (Naito et al., 2020). The extent of ischemic injury in such surgeries is minimized by limiting the ischemic time and packing organs in ice (Naito et al., 2020). However, ischemic injury remains a common complication (Naito et al., 2020; Böttcher et al., 2021).

Reperfusion of an ischemic organ also leads to muscular damage and oxidative stress, often exacerbating myocardial necrosis (Halestrap et al., 2004). Several cardioprotective strategies have been examined in the setting of acute myocardial infarction and cardiac surgery, however at present there are no proven therapies to prevent reperfusion injury.

While IPC reduces both ischemic injury and reperfusion injury, it is clinically impractical, potentially dangerous, and impossible for unanticipated ischemic events. GJA1-20k is translated from an ion channel’s mRNA, yet can protect against I/R injury by inducing protective mitochondrial fission. By mimicking IPC, GJA1-20k has many potential therapeutic targets. For example, GJA1-20k could be administered to organs prior to being harvested for transplantation. As GJA1-20k could be administered systemically, it could potentially protect a variety of organs subjected to ischemic injury during major cardiovascular surgery. GJA1-20k could also be administered in the setting of acute myocardial infarction or stroke to prevent reperfusion injury.

Non-cardiac uses of GJA1-20k are emerging as well. GJA1-20k in astrocytes promotes mitochondrial transport from astrocytes to neurons through full-length Cx43 hemichannels (Ren et al., 2022). This results in increased viability and recovery of neurons after traumatic brain injury, as mitochondrial biogenesis suppresses apoptosis of damaged neurons, suggesting an additional role for GJA1-20k as a protective mitochondrial regulator in the brain (Ren et al., 2020; Shimura and Shaw 2022).

A fascinating aspect of GJA1-20k is that it was discovered in a program that explored the mechanisms of ion channel delivery to limit arrhythmogenesis. Not only is GJA1-20k important to maintain cardiac rhythm, but it has important metabolic and non-cardiac roles as well. We expect that other ion channels have a similar diversity of function. The study of post-translational modification of ion channels can greatly expand our understanding of the full repertoire of ion channel biology.
